# Childhood obesity, metabolic syndrome, and oxidative stress: microRNAs go on stage

**DOI:** 10.1007/s11154-023-09834-0

**Published:** 2023-09-06

**Authors:** Álvaro González-Domínguez, Thalía Belmonte, Raúl González-Domínguez

**Affiliations:** 1 Instituto de Investigación e Innovación Biomédica de Cádiz (INiBICA), Hospital Universitario Puerta del Mar, Universidad de Cádiz, Cádiz, 11009 Spain; 2 Translational Research in Respiratory Medicine, University Hospital Arnau de Vilanova and Santa Maria, IRBLleida, Lleida, Spain; 3 CIBER of Respiratory Diseases (CIBERES), Institute of Health Carlos III, Madrid, Spain

**Keywords:** Childhood obesity, Oxidative stress, miRNA, Metabolic syndrome, Iron metabolism

## Abstract

The incidence of childhood obesity and metabolic syndrome has grown notably in the last years, becoming major public health burdens in developed countries. Nowadays, oxidative stress is well-recognized to be closely associated with the onset and progression of several obesity-related complications within the framework of a complex crosstalk involving other intertwined pathogenic events, such as inflammation, insulin disturbances, and dyslipidemia. Thus, understanding the molecular basis behind these oxidative dysregulations could provide new approaches for the diagnosis, prevention, and treatment of childhood obesity and associated disorders. In this respect, the transcriptomic characterization of miRNAs bares great potential because of their involvement in post-transcriptional modulation of genetic expression. Herein, we provide a comprehensive literature revision gathering state-of-the-art research into the association between childhood obesity, metabolic syndrome, and miRNAs. We put special emphasis on the potential role of miRNAs in modulating obesity-related pathogenic events, with particular focus on oxidative stress.

## Introduction to childhood obesity and metabolic syndrome

Childhood obesity is nowadays a pandemic health issue, affecting over 41 million children under five according to recent estimations from the World Health Organization [1]. Obesity is closely related to various cardiovascular risk factors, such as hyperglycemia, dyslipidemia, and high blood pressure, which altogether constitute the so-called metabolic syndrome (MetS) and represent the main drivers of obesity-related deleterious repercussions over health. Notably, around one third of children with obesity suffer from MetS components, with insulin resistance (IR) being the most prevalent [2]. Nevertheless, unlike the above-defined “metabolically unhealthy obesity” (MUO), part of the population with obesity does not present comorbidities, which is known as “metabolically healthy obesity” (MHO) [3]. In this respect, it is also noteworthy that obesity-related metabolic complications may in turn trigger several other pathologies, such as type 2 diabetes mellitus (T2DM), non-alcoholic fatty liver disease (NAFLD), cardiovascular diseases, and even cancer [2].

Although obesity lacks a concrete etiology, it is known to be the consequence of a complex cluster of interrelated risk factors, including the microbiome, environmental, genetic, perinatal, nutritional, psychosocial, and metabolic factors [4, 5]. In particular, inflammation and oxidative stress (OS) have been described to be tightly interrelated in a vicious cycle that participates in many of the pathological processes behind obesity and related complications [6]. On the one hand, fat accumulation triggers chronic inflammation through several molecular mechanisms, namely immune response activation, cytokine secretion, oxygen flow shrinkage, cellular necrosis, and disturbed lipid homeostasis [7]. In this vein, increased cytokine secretion by adipocytes and subsequent subclinical inflammation is known to promote MetS in subjects with obesity [8]. Cytokines also have a role in the synthesis of acute phase proteins [9] and the invasion of innate immune cells into adipose tissue. Neutrophil infiltration has been proposed as the initial step in the recruitment of macrophages and other immune cells (such as T or B lymphocytes) within adipose tissue. These macrophages in adipose tissue are believed to originate from bone marrow monocytes [10, 11]. While obese fat has large quantities of the pro-inflammatory M1 type of macrophages, lean fat is concentrated in the M2 anti-inflammatory type of macrophages [12]. Eosinophil levels, which are necessary for the maintenance of M2 macrophages, have been reported to be downregulated in obesity [13, 14]. Concurring with these data, hypereosinophilic mice have been found to be protected from IR, whereas mice lacking them develop more body fat, impaired glucose tolerance, and decreased insulin sensitivity [13, 14]. Finally, natural killer T cells can also play relevant roles in adipose tissue inflammation, thereby influencing the susceptibility to develop obesity and IR in a process in which natural killer T cells are influenced and influence the microbiome [15]. Within this proinflammatory environment, activated immune cells liberate reactive oxygen species (ROS) and, when sustained for prolonged time periods, provoke exacerbated OS. After binding their receptors, cytokines can both initiate ROS production and promote the induction of other inflammatory signals. Thus, proinflammatory cytokines such as interferon-𝛾 or IL6, and proinflammatory components such as lipopolysaccharide, have been found to increase nicotinamide adenine dinucleotide phosphate oxidase (NOX)-dependent ROS production [6, 16]. At the same time, the production of ROS may prime signaling cascades that bidirectionally promote proinflammatory gene expression. In this venue, reactive species can lead to inflammation through the activation of protein kinase C, c-Jun-N-terminal kinase, nuclear factor 𝜅B (NF-𝜅B), mitogen-activated protein kinases, or NOD-like receptor protein 3 inflammasome, among others. Along the process of repairing oxidatively damaged DNA, signaling cascades culminating in NF-𝜅B activation are triggered, leading to proinflammatory gene expression. Similarly, OS has been linked to monocyte adhesion to vascular endothelial cells, which also results in NF-𝜅B activation. In human macrophages, a marker of lipid oxidation, 8-isoprostane, is known to activate mitogen-activated protein kinases and lead to increased expression of inflammatory chemokines such as IL-8. Finally, OS mediates NOD-like receptor protein 3 inflammasome activation by means of the dissociation of the thioredoxin-interacting protein/thioredoxin (TRX) complex, thus allowing the interaction between thioredoxin-interacting protein and NOD-like receptor protein 3, and subsequently leading to its activation [6, 16]. Moreover, lipids, proteins, and nucleic acids can be modified under pro-oxidative environments, which may subsequently act as danger-associated molecular patterns (DAMPs) and provoke innate immune responses [17]. Accordingly, childhood obesity and MetS have repeatedly been associated with a sharpened pro-inflammatory milieu (i.e., increased cytokines, disturbed white blood cell counts) [18] and impaired redox metabolism, this latter reflected in reduced content of endogenous and exogenous antioxidants [19] and raised levels of oxidative damage byproducts [20]. In this respect, we have recently demonstrated that depletions in erythroid antioxidant systems are primary hallmarks in the onset of childhood obesity, with MUO children presenting a sharpened pro-oxidative erythroid environment when compared to MHO subjects, as reflected in higher levels of OS byproducts and impaired antioxidant capacity [20, 21].

In this context, studies involving pediatric patients are of major interest to get new insights into the molecular basis behind the onset of obesity at early ages and, thus, to facilitate the development of efficient therapies to prevent further complications. To this end, it is critical to understand the contribution of genetic and epigenetic traits in childhood obesity and its comorbidities. In this review article, we aim to gather state-of-the-art research into the role of micro-ribonucleic acids (miRNAs) in obesity-related pathogenic events, with particular focus on OS.

## An overview on the association between miRNAs and childhood obesity

miRNAs are short (19–23 nucleotides), single-stranded, and non-coding RNA molecules participating in post-transcriptional regulation of genetic expression, which are known to modulate up to 60% of the genes encoded within the human genome [22]. In particular, they act as regulators of messenger RNA (mRNA) degradation and as protein synthesis blockers by binding to untranslated regions (UTRs) of target mRNAs [23]. Nevertheless, recent findings suggest that miRNAs might also up-regulate gene-transcription [24]. To date, ca. 2500 mature miRNAs are registered in the miRbase human database (Release 22.1, October 2018, http://www.mirbase.org/) [25]. As each miRNA is able to target above one hundred genes and, in turn, multiple miRNAs participate in the expression of the same transcript, miRNA dysregulations may provoke profound disturbances in a multitude of biological networks [25]. Although miRNAs modulate genetic expression within cells, they can also be loaded into extracellular vesicles (e.g., exosomes or microvesicles) and released to the circulation, thereby being protected against RNase degradation and allowing cell-to-cell communication. In this venue, miRNAs sorting into extracellular vesicles seems to be a selective process, although the mechanism by which the cells choose miRNAs to be loaded and secreted remains unclear [26]. Interestingly, most body fluids (e.g., blood, breast milk, urine, or saliva) contain exosomes or microvesicles, opening the window to new transcriptomics strategies in biomedical research. Thus, the study of miRNAs has gained great interest in recent years to characterize complex health processes, including obesity and its related syndromes [27].

Many authors have previously delved into the potential role of miRNAs as predictors of obesity development in neonates [28, 29] and as biomarkers of early childhood obesity [30, 31]. In fact, childhood obesity has been described to be accompanied by profound deregulations in the circulating miRNA profile [32]. On the one hand, it has been reported that obese mice adipocytes release more miRNA-containing exosomes compared to lean mice adipocytes [33]. Besides these changes in absolute miRNA contents, obesity is also recognized to be the pathology with the highest percentage of genetic variants in the 3’UTR region of mRNAs, which modulate their interaction with miRNAs [34, 35]. Moreover, *Mansego et al.* proved that several miRNAs coding regions present CpG methylation patterns specific to childhood obesity, pinpointing to a role of epigenetic regulation in obesity development [36]. Transcriptomics techniques have also been widely employed to unravel the association between miRNAs and obesity-related risk factors, including diet, gut microbiota, perinatal conditions, and genetic background.

For instance, higher Mediterranean diet adherence has been related to a switch toward healthier circulating miRNAs profile [37], whereas high-caloric diet consumption leads to increased levels of miRNAs involved in obesity development and progression [38]. Furthermore, miRNAs are known to participate in appetite control in childhood obesity by regulating hormones such as leptin [39] or neuropeptide Y [40]. In this vein, growing evidence supports that miRNAs, diet, and gut microbiota may bidirectionally modulate each other. Thus, miRNA-10a-5p has been proposed to improve high fat diet (HFD)-triggered glucose intolerance and IR through the modulation of the microbiome and its metabolism [41, 42]. External stimuli during fetal development also have great impact on the onset of obesity [4]. Obesity induced by maternal diet negatively impacts offspring body composition in a process that is accompanied by age-dependent alterations in miRNA-582 expression [43]. *Joshi et al.* reported that in utero exposure to maternal obesity provokes sexually dimorphic perturbations in miRNA profiles [44]. Similarly, both paternal HFD and exercise have been described to elicit a sex-specific effect on T2DM risk in offspring by altering sperm miRNA expression [45]. Finally, sex is also known to influence circulating concentrations of some miRNAs in adolescents with obesity [46], which in turn show sexually dimorphic associations with inflammatory biomarkers [47]. This concurs with the general observation that female subjects are more susceptible to weight gain, although men are prone to suffer from obesity-related comorbidities [4]. This could be mainly allocated to sex differences in adipose tissue distribution, as young men normally have higher visceral fat depots, whilst pre-menopausal women accumulate subcutaneous adipose tissue [48, 49]. Indeed, visceral adipose tissue has increased levels of pro-inflammatory macrophages than subcutaneous adipose tissue, so male adults and children have raised content of proinflammatory molecules and diminished inflammation resolution capacity compared to females [50–52].

Numerous authors have also explored the plausible link between miRNAs and a myriad of childhood obesity-related comorbidities, such as MetS [53–55], T2DM [56, 57], NAFLD [58], chronic kidney disease [59], nephropathy [60], endothelial dysfunction [61], colitis [62], or cancer [63, 64]. Interestingly, miRNAs have also shown potential as biomarkers of response to intervention strategies against obesity [65–67]. In this respect, *Liao et al.* proved exercise-based strategies to affect some obesity-related miRNAs in childhood obesity [68]. Also, liraglutide is known to promote the browning of white adipose tissue by downregulating miR-27b expression [69]. Accordingly, several authors hypothesize that personalized therapeutic strategies based on microRNAs administration or inhibition bears promise for treating obesity and metabolic disorders [70–73].

## The involvement of miRNAs in central pathogenic events behind obesity: adipogenesis, insulin metabolism, and inflammatory processes

Childhood obesity is a multifactorial disorder in which a number of closely interrelated pathogenic events participate, namely adipogenesis, insulin metabolism, inflammation, and OS. Thus, understanding the molecular basis underlying these disturbances is a topic of great interest.

Since obesity can primarily be regarded as an abnormal or excessive fat accumulation, altered adipogenesis can be considered as a pivotal player in childhood obesity. In this vein, although most of the molecular pathways involved in adipogenesis are shared between subjects with and without obesity, the onset and progression of obesity have been related to specific miRNA perturbations along this process. Thus, patients with obesity showed a stronger downregulation of miRNAs involved in adipogenesis when compared to lean subjects [74]. As expected, many of the miRNAs that are differentially expressed in visceral adipose tissue of children with obesity have been reported to be enriched in pathways related to lipid metabolism [75]. Some of the most affected pathways at the transcriptomics level by these obesity-related miRNAs have been found to be fatty acid oxidation, ketogenesis, lipogenesis, and lipid uptake [76–78], which could be directly related to increased adipogenesis, fat mass gain, and liver steatosis [79–81]. On the other hand, obesity is also known to hamper some miRNA-mediated protective mechanisms that could modulate adipogenesis [82–84], adipose tissue browning [85, 86], and autophagy inhibition [78].

Obesity and its common comorbidities are also characterized by profound disturbances in insulin homeostasis and related biological processes, such as carbohydrate and lipid metabolisms. Pancreatic β-cells are responsible for sensing glucose levels and mediate insulin secretion in a two-step process. First, glucose enters the β-cell, where it is metabolized in the glycolytic pathway and the tricarboxylic acid cycle to produce adenosine triphosphate (ATP). The increase in cellular ATP levels promotes the closure of ATP-sensitive potassium channels, provoking membrane depolarization and the opening of voltage-dependent calcium channels. The raise in cellular calcium content finally triggers insulin secretion. For the second phase, actin filaments need to be reorganized to accomplish the recruitment of intracellularly stored granules [87–89]. Once released, insulin binds to the α chain of its membrane-located receptor, thus causing structural changes in the β chain thanks to tyrosine kinase mediated auto-phosphorylation of tyrosine residues. Then, phosphorylated receptors recruit intracellular components to initiate signaling pathways. Depending on the tissue and the intracellular substrate, insulin may promote glucose utilization and storage by activating glycolysis, glycogen synthesis, and adipogeneses; by inhibiting gluconeogenesis, lipolysis, and glucagon secretion; or by increasing glucose transport [90]. Within this tangled crosstalk of intertwined processes, miRNAs are recognized to be directly involved in regulating insulin signaling and glucose metabolism at different levels, thereby being capable of promoting either insulin sensitivity [91] or IR [92, 93] in subjects with obesity. In particular, numerous studies have proven the ability of miRNAs to alter carbohydrate metabolism by modulating: (i) insulin transcription and secretion [92], (ii) insulin signaling (e.g., the PI3K-AktmTOR pathway [91, 93], insulin receptor [94], insulin receptor substrates [26], insulin-like growth factor 1 receptor [94]), (iii) glucose transport [26, 93], (iv) gluconeogenesis [95], (v) glycogenesis [96], (vi) glycogenolysis [94], and even (vii) OS-mediated pancreatic β-cell dysfunction and apoptosis [97].

To conclude, a few authors have also described obesity-related miRNA dysregulations to be tightly correlated with a multitude of inflammation biomarkers, such as tumor necrosis factor α (TNFα), interleukin 1 receptor antagonist, IL-8, IL-15, procalcitonin, adiponectin, or C-reactive protein [47, 98, 99]. In this vein, it has recently been demonstrated that the typical inflammatory status present in childhood obesity could modulate miRNA contents in adipocytes. Thus, the expression of miR-424 has been found to be higher in adipose tissue of children with obesity, whereas TNFα can bind to its promoter region and, consequently, decrease its transcription [100]. Interestingly, the inoculation of gut microbiome from children with obesity to mice resulted in the enrichment of colon and liver pro-inflammatory miRNAs, resulting in higher expression of pro-inflammatory markers such as TNFα and IL6 [101]. Furthermore, the above-mentioned raise of circulating cytokines may also mediate acute phase protein production [9] and infiltration of innate immune system cells into adipose tissue. In this context, miRNAs have been proposed as main drivers of immune cell differentiation, and immune cell-derived miRNAs to be involved in the occurrence of obesity-related complications. For instance, miR-150 is known to suppress obesity-related inflammation by modulating B-cell development, activation, and function in adipose tissue [102]. Also, miRNAs can regulate macrophage infiltration rate and switching between pro-inflammatory and anti-inflammatory phenotypes, exerting both protective and harmful effects against obesity-related inflammation and IR [26, 33]. Changes in monocyte’s miRNA cargo have been related to inflammatory action. Thus, obese monocytes have lower levels of miR-146b-5p, an important driver of globular adiponectin’s anti-inflammatory action [103]. Recently, *Macartney-Coxso et al.* showed gastric bypass to lower the circulating levels of miR-223-3p, a miRNA targeting NOD like receptor 3, thereby resulting in reduced adipose concentration of this proinflammatory marker [104].

## Childhood obesity, oxidative stress, and miRNAs

### The molecular basis of oxidative stress

OS is a phenomenon provoked by an imbalanced generation of ROS with respect to the detoxification capacity of antioxidant defenses [6, 16]. On the one hand, reactive species may have an endogenous (e.g., cyclooxygenase, COX; Fenton reaction; glucose autooxidation; NOX; peroxisomes; uncoupling of nitric oxide synthase, NOS) or exogenous (e.g., bacteria, cigarette smoking, medications, industrial chemicals, ozone, X-rays) origin [105]. Under situations of ROS overproduction, biomolecules may suffer modifications that cause their degradation or inactivation. In particular, post-translational modifications (PTMs) of proteins are relevant OS-derived cellular damages that affect protein lifespan, protein-protein interactions, protein solubility, and enzyme function [106]. Among them, protein glycosylation is one of the most abundant PTMs regulating the proteome and can be expressed in different forms (e.g., O-glycosylation, N-glycosylation, or O-GlcNAcylation) [107]. Moreover, ROS can also activate autophagy by modulating the PI3K-Akt-mTOR axis, AMP-activated protein kinase (AMPK), or forkhead box transcription factor O (FoxO) [108].

To face such stressful situations, the organism disposes of a well-organized barrier of antioxidant defenses, which comprises a number of stable molecules capable of neutralizing free radicals to minimize toxic effects and cellular damage. This antioxidant system is composed by endogenous (e.g., TRX; glutathione, GSH; α-lipoic acid, melatonin, coenzyme Q10, albumin, uric acid, ferritin) and exogenous (e.g., ascorbic acid, α-tocopherol, carotenoids, polyphenols, trace elements) compounds, as well as by various antioxidant enzymes [109]. Antioxidant enzymes can in turn be divided into primary enzymes, when they act directly in scavenging ROS, or secondary enzymes, when their role is to support the action of endogenous non-enzymatic antioxidants. The most important primary antioxidant enzymes are superoxide dismutases (SODs), catalase (CAT), glutathione peroxidases (GPX) and peroxiredoxins (PRDXs). SODs are metalloenzymes responsible for the detoxification of superoxide radicals into H_2_O_2_. Then, the hydrogen peroxide produced by SODs must be detoxified by peroxidases. To this end, PRDXs encompass different isoforms with one or two redox-active cysteine residues. The reactivation of PRDXs is accomplished by using TRX as reducing agent. On the other hand, GPXs are selenium-dependent oxidoreductases that use GSH as the electron donor. Finally, CAT is a heme group-containing enzyme composed by four monomers. Although CAT does not require GSH or TRX as electron donors, its activity is dependent on nicotinamide adenine dinucleotide phosphate (NADPH) as a reducing power source. Therefore, reducing power generation by secondary antioxidant enzymes is required for a correct function of antioxidant enzymes. Together with isocitrate dehydrogenase (IDH), which mediates NADPH recycling in the mitochondria, glucose-6-phosphate (G6PDH) and 6-phosphogluconate (6PGDH) dehydrogenases are the main sources of cellular reducing power through the pentose phosphate pathway (PPP). This NADPH can in turn be used for GSH and TRX reduction by the action of reductases, such as glutathione reductase (GSHR) and thioredoxin reductase (TRXR) [109, 110].

In this context, several signaling pathways may participate in antioxidant defense modulation. Sirtuins (SIRT) are involved in sensing and regulating redox status in cells, exerting a protective effect against oxidative stressors. SIRTs are able to deacetylate other proteins that participate in response against cell stress, such as FoxO transcription factors, NF-𝜅B, or nuclear factor E2-related factors (Nrf) [111]. In the absence of ROS, Kelch-like ECH-associated protein 1 (Keap1) binds to Nrf2 and triggers its degradation. Nevertheless, Keap1 is oxidized in the presence of ROS, which prevents its binding to Nrf2. Once in the nucleus, Nrf2 activates genes of the antioxidant system [112]. On the other hand, the receptors for advanced glycation end products (RAGE) and toll-like receptors (TLRs) activate NF-𝜅B, which may exert both anti- and pro-oxidant roles by targeting manganese-SOD, ferritin heavy chain, heme-oxygenase 1 (HO1), GPx, or NOX [113–115]. Finally, peroxisome proliferator activated receptors (PPARs) can heterodimerize with retinoid X receptors (RXR) to bind PPAR-responsive regulatory elements (PPRE), thereby regulating gene expression [116]. Peroxisomes also contain different ROS generating and scavenging enzymes, and their size and enzymatic availability is influenced by PPARs and inflammation [117].

### Background on the association between childhood obesity and oxidative stress

Childhood obesity and MetS are well-known to be characterized by increased circulating and cellular levels of ROS and OS byproducts, together with significant perturbations in multiple antioxidant systems. We have recently demonstrated that children with obesity and concomitant IR exhibit compromised erythroid antioxidant defenses after undergoing an oral glucose tolerance test (OGTT), the most used technique for the diagnosis of metabolic impairments [118]. When facing this stressful situation caused by glucose overload, MUO children display an exacerbated oxidative milieu, as mirrored by an impaired redox status (e.g., altered GSH/GSSG, NADP/NADPH) and increased levels of erythroid malondialdehyde (MDA) and carbonyl groups [20]. Similarly, chronic overnutrition leads to persistently increased blood glucose, which is toxic for our organism by generating free radicals (i.e., glucotoxicity) [119]. Under this scenario, proteins are expected to suffer from glycosylation, although we recently found children with obesity and IR to have decreased rates of catalase O-GlcNAcylation, a reaction that is mediated by O-linked N-acetylglucosamine transferase (OGT) [21]. In this line, high monosaccharide concentrations also provoke glycation of other biomolecules and, consequently, result in the overproduction of pro-oxidative mediators, especially advanced glycation end-products (AGEs) [120, 121]. In turn, AGEs interaction with its receptor triggers the activation of NOX, which is also activated under proinflammatory conditions in a process that is mediated by protein kinase C [122–124]. Moreover, AGEs may also mediate NF-kB up-regulation [113, 114]. Conversely, SIRTs, Nrf2, PPAR-γ, and activated AMPK expressions have been found to be diminished in children with obesity and metabolic impairments [125–127]. In this respect, *Gastaldi et al.* described that weight loss results in upregulated expression of peroxisome proliferator-activated receptor gamma coactivator-1 (PGC-1α), thus contributing to the improvement of insulin sensitivity [128].

As expected, the above-mentioned oxidative disturbances behind obesity and MetS are normally accompanied by extensive dysregulations in concentrations and activities of various antioxidant enzymes. In a study performed in 2018, although no differences were described in serum SOD activity between subjects with normal and high body fat, a depleted activity was found when concomitant MetS was present [129]. However, data regarding SOD activity in children with obesity are contradictory, since it has been described to be both increased and decreased, as reviewed by *Codoñer-Franch et al.* [130]. In this line, we reported that CAT, GSHR, and GPx could be the antioxidant enzymes that are majorly affected by IR in prepuberal children with obesity. This was accompanied by a blunted capacity of reducing power generation through the PPP, as reflected in diminished G6PDH and 6PGDH activities along an OGTT [20]. This concurs with previous studies describing that mitochondrial NADPH production by IDH2 protects mice from HDF-induced OS [131].

### The involvement of miRNAs in obesity-related oxidative stress

**Fig. 1:**
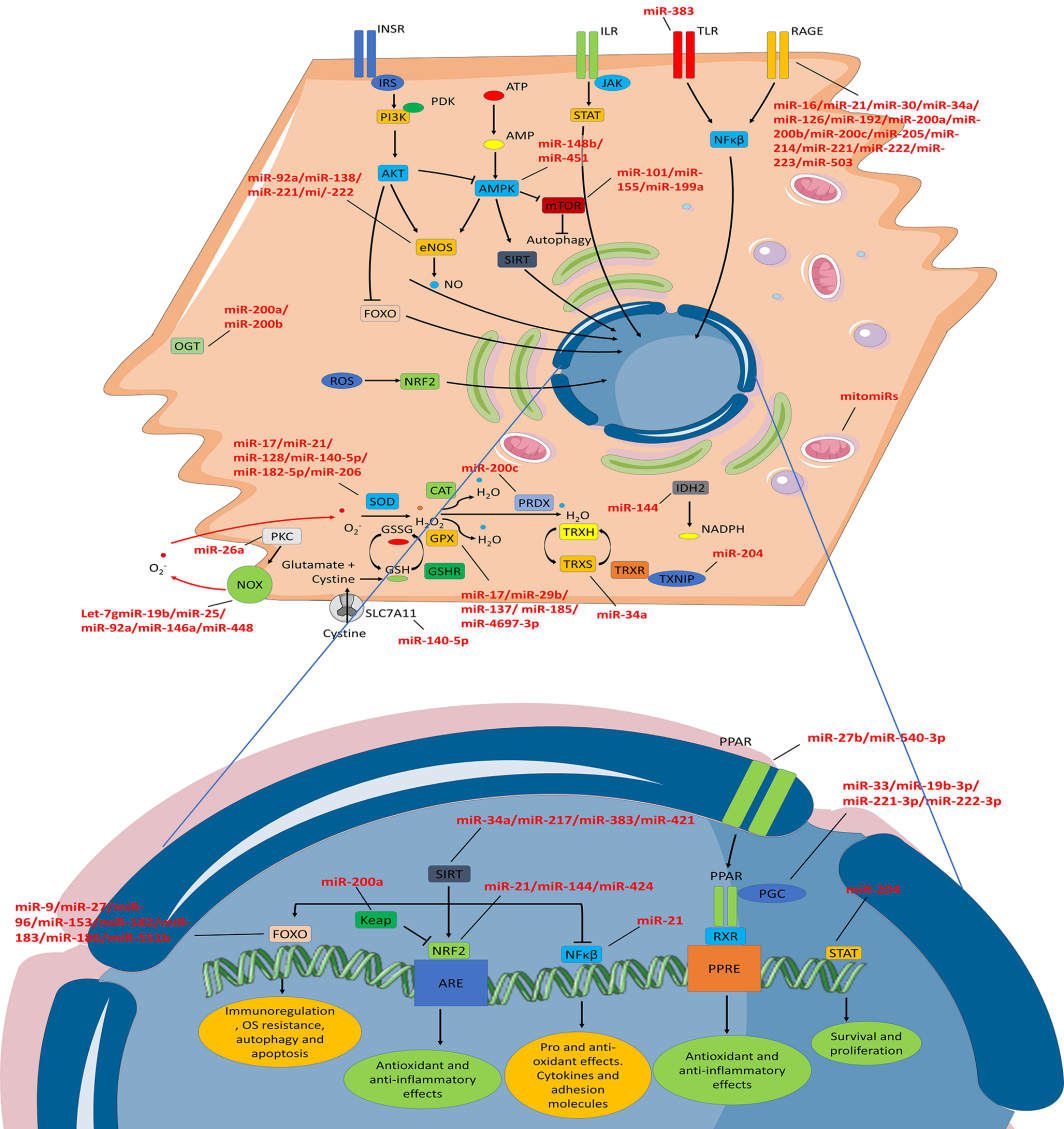
Overview of the main miRNAs involved in oxidative stress response in obesity and related comorbidities

Among many other mechanisms, miRNAs seem to play a bidirectional role in the onset of the characteristic OS exacerbation that is observed in childhood obesity and MetS. On the one hand, the expression and secretion of miRNAs may be affected by various sources of ROS, and dysregulated miRNAs can in turn influence the expression and activity of antioxidant defenses (Fig. 1) [132–134]. Moreover, miRNAs suffer from oxidative modifications that lead to mRNA target misrecognition, a process that has previously been related to the development of cardiac hypertrophy and initiation of apoptotic events in cardiac cells [135–137]. Additionally, it is noteworthy that obesity is characterized by lower mitochondrial key gene expression and abundance. In this venue, mitochondria-located miRNAs (mitomiRs) are main regulators of mitochondrial function and adipogenesis, being involved in hyperlipidemia and hyperglycemia-induced mitochondrial dysfunction through the modulation of its fusion-fission, mitophagy, or even thermogenesis [138, 139]. Furthermore, miRNAs participate in endoplasmic reticulum (ER) stress generation by disturbing central metabolic pathways, thus leading to the characteristic hyperlipoproteinemia that is observed in MetS and affecting proadaptive or proapototic pathways. Similarly, altered miRNA expression has been linked to ER stress induction by nutrient oversupply [140].

Regarding oxidative damage, many obesity-related miRNAs have been described to target several of the above-mentioned mechanisms of ROS production. For instance, it has been reported the ability of miR-140-5p, miR-221-3p and miR-182-5p to lower ROS, MDA, and oxidized low-density lipoprotein (LDL) in cellular models of atherosclerosis by targeting TLR4 or metalloproteinase domain-containing protein 22 [141–143]. Similarly, miR-200a and miR-200b control protein PTMs by degrading OGT mRNA, although their levels are diminished under hyperglycemic states, as well as by modulating endothelial inflammation under conditions of high circulating glucose [144]. On the other hand, COX2 and endothelial NOS are predicted targets of miR-6796-5p/miR-4697-3p and miR-92a/miR-221/miR-222, respectively, which are known to be upregulated in MUO subjects, thereby pointing to a plausible role of these miRNAs in metabolic disease prevention in patients with obesity through OS reduction [145–147]. High glucose and AGEs levels have been described to repress miR-126, a miRNA with proven protective effect over endothelial progenitor cells, thus resulting in increased generation of proinflammatory cytokines and ROS [148]. In contrast, miR-34a mediates AGEs-induced apoptosis of endothelial progenitor cells. In fact, some drugs improve endothelial function and regenerative capacity of damaged diabetic endothelial cells by inhibiting miR-34a [149]. [33] Furthermore, many miRNAs affected by glucose and cholesterol levels have been found to directly modulate NOXs protein levels and activity, leading to higher superoxide levels and oxidative/nitrative stress [61, 144, 150, 151]. Finally, a number of obesity-related miRNAs are also capable of regulating AMPK and mammalian target of rapamycin (mTOR), which trigger ROS-induced autophagy [61].

To conclude, it is worth mentioning that miRNAs may serve as master regulators of antioxidant enzyme expression and activity in obesity as well. They can indirectly affect their expression by modulating SIRTs, Nrf, PPARs, PGC-1α, FokO, TLRs, Keap1, and NF-𝜅B [33, 61, 97, 146, 152–159], but also modify oxidative metabolism by directly targeting specific antioxidant enzymes. Thus, miR-34a, miR-217, and miR-383, which are upregulated in atherosclerotic lesions, obesity, and diabetes, are known to target SIRT1, which in turn is an important regulator of metabolic disorders by promoting eNOS transcription and activity [33, 146, 153, 155]. As described by *Kong et al.*, under hyperglycemic states, long non coding RNAs may act as miRNAs sponges, buffering their effect and reverting OS and cell damage [154]. Similarly, miR-221/222 and miR-33 exert pro-atherogenic effects by targeting PGC-1α and altering mitochondrial biogenesis and OS [146]. In response to glucose oscillations, miR-21 also affects ROS generation by targeting FoxO [152]. Complementarily, miRNAs can play an important role in the pathophysiology behind metabolic disorders by regulating PPARs. Upon inflammatory stimuli, miR-27b lead to PPARγ mRNA destabilization [156, 157]. On the other hand, scientific evidence points to a pivotal role of miRNAs in controlling the expression of SODs and GPxs in obesity. Obesity has been shown to influence SOD expression in a process in which miR-17 and miR-21 take part. In other studies, miR-17, miR-29b, miR-137, and miR-185 have been described to target several GPx isoforms in an adipogenesis-independent process that is regulated by circulating glucose levels [160–162]. Nevertheless, works assessing the impact of miRNAs over the expression of other antioxidant enzymes are scarce. PRDX2 has been described to be downregulated by miR-200c, which is upregulated in obese subjects and implicated in diabetes-related endothelial dysfunction [61]. Also, miR-34a overexpression leads to OS in obesity-related NAFLD by lowering TRX levels [146]. Similarly, miR-204 also participates in TRX downregulation in a mechanism involving TRX-interacting protein, responsible for TRX inhibition [163]. Finally, it is also recognized that miRNAs control reducing power generation by affecting the levels and activities of G6PDH, 6PGDH, and IDH [164–167]. In this line, miR-1, miR-206, and miR-613 have been proposed as a therapeutic target for the treatment of different types of cancer thanks to their ability to downregulate G6PDH and 6PGDH [164, 165].

### Antioxidant-based therapeutic strategies in the management of obesity-related complications

The scientific community has made great efforts in the search of successful antioxidant-based therapies for the treatment of a wide variety of diseases. Thus, strategies based on increasing the synthesis of antioxidant enzymes, ROS removal, increase of antioxidant species using precursors, inhibition of ROS sources, use of dietary antioxidants, or inhibition of redox signaling have extensively been explored [168]. As reviewed by Wang et al., antioxidant supplementation may have positive effects on several indicators of obesity (BMI, HOMA-IR, or fasting blood glucose) and related components such as antioxidant capacity (MDA or SOD), inflammatory biomarkers (TNFα), and lipid metabolism (total cholesterol, triglycerides, or LDL) [169].

On the basis of the above-mentioned rationale linking OS to an altered regulation of miRNAs, various authors have also investigated the utility of restoring the miRNA profile as a candidate therapeutic tool to prevent obesity-related OS. In this venue, *Cannataro et al.* assessed the changes in miRNAs that a ketogenic diet could exert in a population with obesity. They found that the dietary intervention induced a lean-like miRNA profile, which was accompanied by a switch into a better oxidative control [170]. Indeed, the consumption of antioxidant compounds such as polyphenols may target specific miRNAs related to OS in obesity and modulate their activity (e.g., by influencing miRNAs functionality through alterations in their binding capacity to the target mRNA, or by regulating their biogenesis process), consequently reducing the risk of developing chronic diseases [171, 172]. Specifically, resveratrol supplementation in patients with hypertension has been proved to result in improved inflammatory profiles by means of the modulation of miR-21, miR-155, and miR-34a. Many other antioxidant compounds, such as pterostilbene, carnosic acid, and melatonin, also appeared to downregulate miR-34a in fructose fed rats and HFD mice, leading to restoration of SIRT1 activity, inhibition of lipogenic activity, alleviated dyslipidemia, and anti-apoptotic effects [146]. Also, curcumin and polydatin restore the expression of miR-200a, thus reducing inflammasome activation and resulting in higher Nrf2-dependent antioxidant defense through miR-200a-mediated regulation of Keap1 [146]. Lycopene supplementation improved hepatic steatosis in HFD mice by restoring miR-21 levels, which in turn downregulates fatty acid-binding protein 7 and reduces intracellular lipid accumulation in cultured hepatic cells [173]. Finally, the anti-obesogenic effect of garlic seems to be, at least in part, mediated by diallyl trisulfide, whose oral administration in HFD rats reduced both triglyceride levels and white adipose tissue weight gain in a process accompanied by miR-335 inhibition and decreased levels of lipogenic mRNAs [174]. Nevertheless, it should be noted here that most works studying the effect of antioxidant compounds over miRNAs profile have been carried out in vitro using high concentrations, rather than using in vivo approximations with metabolites at low concentrations in the circulation [175].

Despite all these efforts, antioxidant strategies currently face several limitations. Almost all of them are non-specific strategies that may affect other essential pathways. In addition, OS is normally a secondary agent in the development of diseases, and not the primary cause, so addressing this problem usually has no beneficial effect on the pathogenesis of the disease. Moreover, the high affinity of cellular components for reactive species limits the usefulness of mimetics, with lower chelating capacity. In this line, the agents commonly used in antioxidant strategies may not reach effective concentrations in the body for different reasons, such as their low half-life [168]. In conclusion, deeper knowledge of the molecular bases underlying OS is needed to develop successful therapeutic strategies.

### Childhood obesity, Iron Metabolism, and miRNAs

Metals and metalloid elements can regulate oxidative metabolism though different mechanisms, either by generating ROS via redox cycling reactions (redox-active metals, e.g., iron, copper), by depleting endogenous antioxidant levels (redox-inactive metals, e.g., cadmium, mercury), and by directly contributing to the antioxidant defense (e.g., selenium, manganese). Accordingly, disruptions in metal metabolism may provoke excessive ROS/RNS production, with subsequent oxidative damage in lipids, proteins, and DNA [176]. In this regard, some studies have previously reported a close link between childhood obesity and metal blood levels [177–179]. Interestingly, we found that metal disturbances are tightly inter-related to the typical hallmarks behind childhood obesity and comorbidities, namely OS, inflammation, impaired insulin metabolism, and dyslipidemia, and in turn can be modulated by different risk factors [177, 180–183]. In particular, growing evidence suggest that childhood obesity and MetS could be related to profound iron metabolism dysregulations at multiple levels, including absorption, storage, transport, utilization, and recycling, as recently reviewed [184]. First, childhood obesity and MetS have been reported to impact iron/heme absorption and assimilation by enterocytes, as well as iron transfer into the circulation. Furthermore, proteins involved in iron transport and storage, like transferrin receptor, nuclear receptor coactivator (NCOA) 4 or ferritin, seem to be also affected in obesity. Similar alterations have been described in other proteins involved in iron recycling, such as haptoglobin or hemopexin, which may have important health consequences considering that most of the daily required iron is obtained though recycling mechanisms. Finally, obesity is also known to impair iron homeostasis by affecting several other pathways, such as the hepcidin-hemojuvelin axis or hypoxia inducible factors [184].

**Fig. 2:**
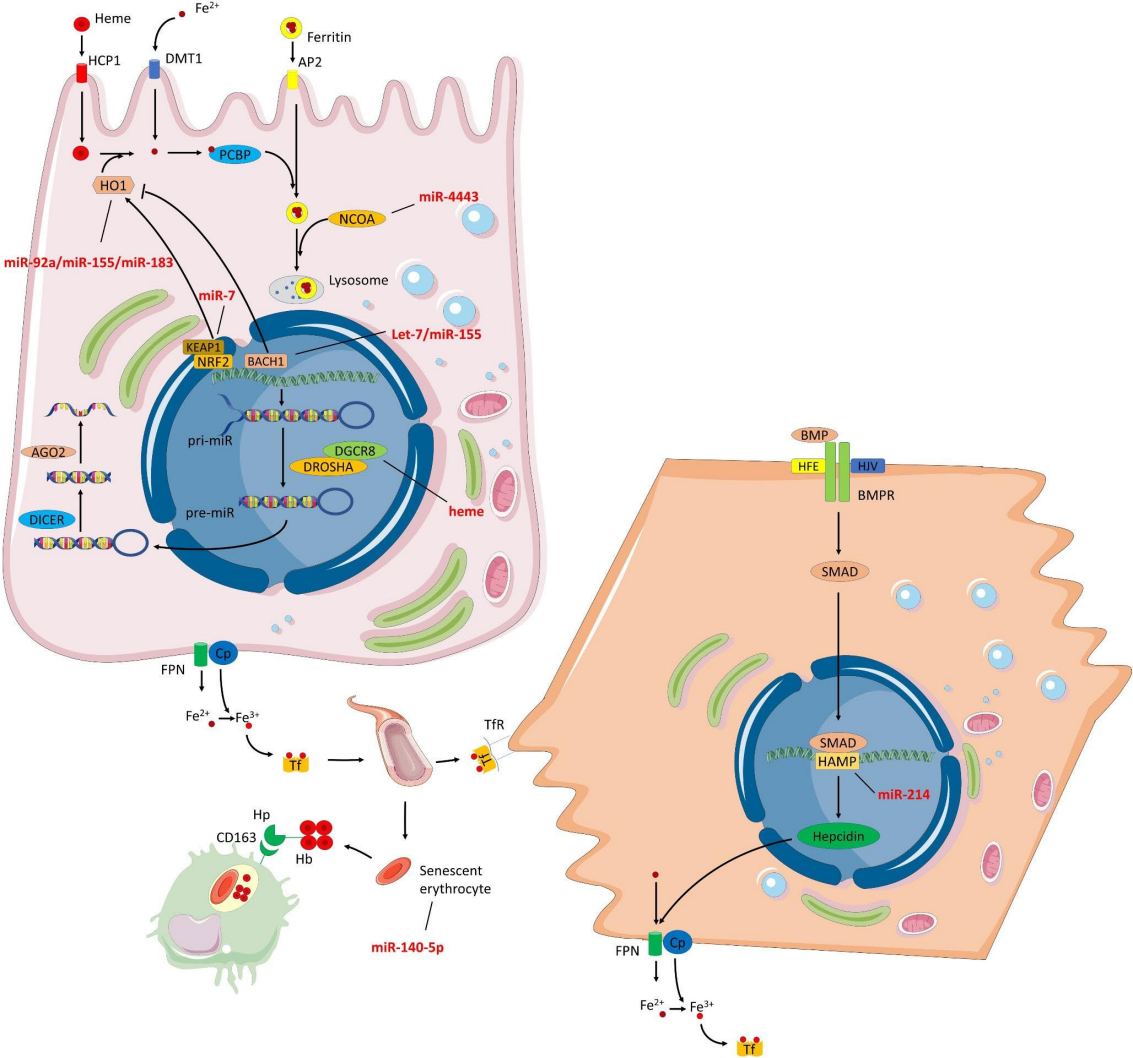
Overview of the main miRNAs involved in iron metabolism regulation in obesity and related comorbidities

Although the involvement of miRNAs in obesity-associated OS has been investigated relatively often, their implication in regulating iron metabolism remains quite unexplored. In this respect, it is nowadays recognized that the relationship between miRNAs and iron is bidirectional, since miRNAs modulate iron metabolism and, at the same time, the different biomolecules participating in iron metabolism also affect miRNA production and expression (Fig. 2). In fact, the RNA-binding protein DiGeorge Critical Region 8 (DGCR8), cofactor of the ribonuclease DROSHA and crucial player in processing miRNAs primary transcripts (pri-miRNA), constitutes a highly active complex when reacting with ferric heme, whereas its reduction into ferrous heme leads to impaired activity [185–187]. Hemin, a heme group byproduct, also enhances the interaction between DGCR8 and the pri-miRNA [188]. On the other hand, miR-374a has been linked to the control of iron overload-induced ROS production, thereby inhibiting iron-induced release of cytokines and limiting hepatic stellate cell activation in fibrotic processes [189]. Some miRNAs have also been related to the development of ferroptosis, a newly described programmed cell death dependent on iron. Indeed, miR-140-5p, which is overexpressed in exosomes from obese adipose tissue-derived macrophages, promotes ferroptosis in cardiomyocytes by impeding GSH synthesis [190]. Furthermore, besides controlling hemolysis, miRNAs also modulate erythropoiesis. Thus, lipopolysaccharide-induced inflammation has been demonstrated to induce miR-122 secretion in mice, which affects erythropoiesis by reducing erythropoietin levels, which enables establishing a link between inflammation-related anemia and miRNAs [191].

Various studies have also evidenced a close link between miRNAs and iron absorption. Iron can be absorbed by enterocytes both in free form and bound to ferritin or to heme groups. Once absorbed by the heme carrier protein 1, heme groups may be either degraded by HO1, which mediates anti-inflammatory, antioxidant, and antiapoptotic effects, or directly absorbed into the circulation. To this end, the regulation of HO1 translation is mediated by antioxidant-response elements, which can be modulated both in a repressive and an inductive way through BTB domain and CNC homolog 1 (BACH1) and Nrf2 transcription factors, respectively. In this context, it has been reported that BACH1 mRNA is inhibited by miR-155 and let-7, thereby inducing HO1 expression in a cytokine-triggered mechanism dependent on NF-𝜅B, stablishing a cytoprotective process to face inflammation [192, 193]. Conversely, miR-7 exerts positive effects over Nrf2 by targeting Keap1 and, consequently, over HO1 levels, leading to reduced intracellular content of hydroperoxides and higher levels of reduced glutathione [194]. Also, miR-92a, which is induced by oxidized-LDL and AGEs, inhibits HO1 expression and impairs endothelial function in diabetic mice, and its suppression ameliorates OS and improves endothelial function [195]. Finally, insulin also seems to exert regulatory effects over HO1 by downregulating miR-155 and miR-183, which are predicted to target HO1 in adipocytes, consequently resulting in higher HO1 expression in a dose-dependent manner [196]. Besides the above-mentioned mechanisms, enterocytes can also absorb dietary ferritin via endocytosis in a process that is mediated by the adaptor-related 2 protein complex. In the cell, NCOA binds ferritin to be delivered to lysosomes for further degradation and iron release [184]. Interestingly, treatment with leptin and insulin has been found to result in miR-4443 overexpression in colorectal cancer cells, which targets and downregulates NCOA1 and TNF receptor associated factor 4. This tumor-suppressive effect is lost in insulin/leptin resistant models (e.g., obesity-induced models), which predisposes to the development of cancer [197].

To conclude, a few authors have also explored the involvement of miRNAs in regulating hepcidin-mediated iron homeostasis. Hepcidin is the main regulator of iron metabolism by degrading ferroportin, a cellular iron exporter. To accomplish its expression, hemojuvelin modulates the binding between the bone morphogenic protein (BMP) and its receptor, establishing a complex that activates small-mothers-against-decapentaplegic proteins (SMADs) and promotes hepcidin antimicrobial peptide (HAMP) gene translation [184]. In this sense, it is well established the potential role of SMAD protein in mediating miRNAs biosynthesis through transcriptional and post-transcriptional mechanisms [198]. Hepcidin expression is also modulated by saturated fatty acids in a process in which miR-214 is involved. Thus, palmitic acid may mediate miR-214 overexpression in HepG2 cells, and miR-214 in turn was described to increase HAMP mRNA levels [199].

## Concluding remarks

The prevalence of childhood obesity and associated disorders, such as type 2 diabetes mellitus and cardiovascular diseases, has grown at a frenetic pace in the last years. Nowadays, these medical conditions have reached pandemic levels and represent an important socio-economic burden in developed countries. Accordingly, the discovery of efficient approaches for diagnosing and treating childhood obesity and related complications has become an urgent need for public health systems. In this respect, oxidative stress is well-known to be one of the most relevant molecular drivers behind these metabolic disorders, within a complex crosstalk involving other intertwined pathogenic events, such as inflammation, insulin disturbances, and dyslipidemia factors. Therefore, the proper regulation of oxidative metabolism has been proposed as a plausible preventive and therapeutic strategy for managing obesity. However, the use of antioxidant molecules in clinical trials has demonstrated limited efficacy up to date, which highlights the need of getting deeper insights into the molecular mechanisms behind obesity-related oxidative stress.

In the last decades, the study of miRNAs has gained great interest for deciphering complex processes in health and disease, including obesity and related comorbidities. Indeed, as each miRNA may target more than one hundred genes, slight dysregulations in their expression might cause profound impairments in a wide range of biological processes. Thus, many authors hypothesize that miRNA profile restoration to normal ranges can be regarded as a powerful therapeutic tool to fight against oxidative damage. In this work, we provide a comprehensive literature revision to delve into the current knowledge about miRNAs dysregulation in childhood obesity and metabolic syndrome. In particular, we have focused on revising the potential role that these non-coding RNAs might play on modulating the characteristic pathogenic hallmarks occurring in childhood obesity, with special emphasis on oxidative stress.

## Abbreviations

MetS: metabolic syndrome
IR: insulin resistance
INSR: insulin receptor
IRS: insulin receptor substrate
MUO: metabolically unhealthy obesity
MHO: metabolically healthy obesity
T2DM: type 2 diabetes mellitus
NAFLD: non-alcoholic fatty liver disease
OS: oxidative stress
ROS: reactive oxygen species
mRNA: messenger RNA
HFD: high fat diet
ATP: adenosine triphosphate
TNFα: tumor necrosis factorα
COX: cyclooxygenase
NOX: nicotinamide adenine dinucleotide phosphate oxidase
NOS: nitric oxide synthase
PTMs: post-translational modifications;
AMP: adenosine monophosphate
AMPK: AMP-activated protein kinase
FoxO: forkhead box transcription factor
TRX: thioredoxin
TRXS: oxidized thioredoxin
TRXH: reduced thioredoxin
GSH: reduced glutathione
GSSG: oxidized glutathione
SODs: superoxide dismutases
CAT: catalase
GPX: glutathione peroxidases
PRDXs: peroxiredoxins
NADPH: nicotinamide adenine dinucleotide phosphate
IDH: isocitrate dehydrogenase
G6PDH: glucose-6-phosphate dehydrogenase
6PGDH: 6-phosphogluconate dehydrogenase
PPP: pentose phosphate pathway
GSHR: glutathione reductase
TRXR: thioredoxin reductase
SIRT: sirtuin
NF-𝜅B: nuclear factor 𝜅B
Nrf: nuclear factor E2-related factors
Keap1: Kelch-like ECH-associated protein 1
RAGE: receptors for advanced glycation end products
TLRs: toll-like receptors
HO1: heme-oxygenase 1
PPARs: peroxisome proliferator activated receptors
RXR: retinoid X receptor
PPRE: PPAR-responsive regulatory elements
OGTT: oral glucose tolerance test
MDA: malondialdehyde
OGT: O-linked N-acetylglucosamine transferase
AGEs: advanced glycation end-products
PGC-1α: proliferator-activated receptor gamma coactivator-1
mitomiRs: mitochondria-located miRNAs
ER: endoplasmic reticulum
LDL: low-density lipoprotein
mTOR: mammalian target of rapamycin
NCOA: nuclear receptor coactivator;
DGCR8: DiGeorge Critical Region 8
BACH1: BTB domain and CNC homolog 1
SMADs: small-mothers-against-decapentaplegic proteins
HAMP: hepcidin antimicrobial peptide
AKT: AKT serine/threonine kinase
ARE: antioxidant response element
eNOS: endothelial nitric oxide synthase
ILR: interleukin receptor
JAK: Janus kinase; PI3K, phosphoinositide 3-kinase
PDK: pyruvate dehydrogenase kinase
STAT: signal transducer and activator of transcription
SLC7A11: solute carrier family 7 member 11
AP2: adaptor-related protein complex 2
AGO2: argonaute 2
BMP: bone morphogenic protein
BMPR: bone morphogenic protein receptor
Cp: ceruloplasmin
CD163: cluster of differentiation 163
DMT1: divalent metal transporter 1
FPN: ferroportin;
Hp: haptoglobin
HCP1: heme carrier protein 1
HO1: heme oxygenase 1
HFE: hemochromatosis protein
Hb: hemoglobin
HJV: hemojuvelin
PCBP: poly(rC)-binding protein
Tf: transferrin
TfR: transferrin receptor

## References

[1] World Health Organization. - Childhood Obesity n.d. https://www.who.int/dietphysicalactivity/childhood/en/ (accessed November 15, 2022).

[2] di Cesare M, Sorić M, Bovet P, Miranda JJ, Bhutta Z, Stevens GA (2019). The epidemiological burden of obesity in childhood: a worldwide epidemic requiring urgent action. BMC Med.

[3] Ahmad MN, Zawatia AA (2021). Current prospects of metabolically healthy obesity. Obes Med.

[4] Han JC, Lawlor DA, Kimm SY (2010). Childhood obesity. The Lancet.

[5] Geng J, Ni Q, Sun W, Li L, Feng X (2022). The links between gut microbiota and obesity and obesity related diseases. Biomed Pharmacother.

[6] Biswas SK (2016). Does the interdependence between oxidative stress and inflammation explain the antioxidant Paradox?. Oxid Med Cell Longev.

[7] Yang F, Dawes P, Leroi I, Gannon B (2018). Measurement tools of resource use and quality of life in clinical trials for dementia or cognitive impairment interventions: a systematically conducted narrative review. Int J Geriatr Psychiatry.

[8] Lancha Junior L, Junior. Pereira-Lancha. Obesity: considerations about etiology, metabolism, and the use of experimental models. Diabetes Metab Syndr Obes 2012:75. 10.2147/DMSO.S25026. 10.2147/DMSO.S25026 PMC3346207 22570558

[9] Purdy JC, Shatzel JJ (2021). The hematologic consequences of obesity. Eur J Haematol.

[10] Kawai T, Autieri M, Scalia R (2021). Adipose tissue inflammation and metabolic dysfunction in obesity. Am J Physiology-Cell Physiol.

[11] Rohm T, Meier DT, Olefsky JM, Donath MY (2022). Inflammation in obesity, diabetes, and related disorders. Immunity.

[12] Liu Y, Xu D, Yin C, Wang S, Wang M, Xiao Y (2018). IL-10/STAT3 is reduced in childhood obesity with hypertriglyceridemia and is related to triglyceride level in diet-induced obese rats. BMC Endocr Disord.

[13] Calco GN, Fryer AD, Nie Z (2020). Unraveling the connection between eosinophils and obesity. J Leukoc Biol.

[14] Daryabor G, Kabelitz D, Kalantar K (2019). An update on immune dysregulation in obesity-related insulin resistance. Scand J Immunol.

[15] Satoh M, Iwabuchi K. Role of natural killer T cells in the development of obesity and insulin resistance: insights from recent progress. Front Immunol 2018;9. 10.3389/fimmu.2018.01314. 10.3389/fimmu.2018.01314 PMC6004523 29942311

[16] Chatterjee S. Oxidative Stress, Inflammation, and Disease. Oxidative Stress and Biomaterials, Elsevier; 2016, p. 35–58. 10.1016/B978-0-12-803269-5.00002-4.

[17] Lugrin J, Rosenblatt-Velin N, Parapanov R, Liaudet L (2014). The role of oxidative stress during inflammatory processes. Biol Chem.

[18] Mărginean CO, Meliţ LE, Huțanu A, Ghiga DV, Săsăran MO (2020). The adipokines and inflammatory status in the era of pediatric obesity. Cytokine.

[19] Stenzel A, Carvalho R, Jesus P, Bull A, Pereira S, Saboya C (2018). Serum antioxidant Associations with metabolic characteristics in metabolically healthy and unhealthy adolescents with severe obesity: an observational study. Nutrients.

[20] González-Domínguez Á, Visiedo F, Domínguez-Riscart J, Ruiz-Mateos B, Saez-Benito A, Lechuga-Sancho AM (2021). Blunted reducing Power Generation in Erythrocytes contributes to oxidative stress in Prepubertal obese children with insulin resistance. Antioxidants.

[21] González-Domínguez Á, Visiedo F, Domínguez-Riscart J, Durán-Ruiz MC, Saez-Benito A, Lechuga-Sancho AM (2022). Catalase post-translational modifications as key targets in the control of erythrocyte redox homeostasis in children with obesity and insulin resistance. Free Radic Biol Med.

[22] Friedman RC, Farh KKH, Burge CB, Bartel DP (2009). Most mammalian mRNAs are conserved targets of microRNAs. Genome Res.

[23] Bartel DP, Metazoan, MicroRNAs (2018). Cell.

[24] Maligianni I, Yapijakis C, Bacopoulou F, Chrousos G (2021). The potential role of Exosomes in child and adolescent obesity. Children.

[25] Kozomara A, Birgaoanu M, Griffiths-Jones S, MiRBase (2019). From microRNA sequences to function. Nucleic Acids Res.

[26] Kim Y, Kim O-K (2021). Potential roles of Adipocyte Extracellular vesicle–derived miRNAs in obesity-mediated insulin resistance. Adv Nutr.

[27] Landrier JF, Derghal A, Mounien L. MicroRNAs in obesity and related metabolic Disorders. Cells 2019;8. 10.3390/cells8080859. 10.3390/cells8080859 PMC6721826 31404962

[28] Marcondes JP, de Andrade C, Sávio PFB, Silveira ALV, Rudge MAD, Salvadori MVC (2018). BCL2 and miR-181a transcriptional alterations in umbilical-cord blood cells can be putative biomarkers for obesity. Mutat Research/Genetic Toxicol Environ Mutagen.

[29] López P, Castro A, Flórez M, Miranda K, Aranda P, Sánchez-González C, et al. miR-155 and miR-122 expression of Spermatozoa in obese subjects. Front Genet. 2018;9. 10.3389/fgene.2018.00175. 10.3389/fgene.2018.00175 PMC5986881 29896216

[30] Ouyang S, Tang R, Liu Z, Ma F, Li Y, Wu J (2017). Characterization and predicted role of microRNA expression profiles associated with early childhood obesity. Mol Med Rep.

[31] Takatani R, Yoshioka Y, Takahashi T, Watanabe M, Hisada A, Yamamoto M (2022). Investigation of umbilical cord serum < scp > miRNAs associated with childhood obesity: a pilot study from a birth cohort study. J Diabetes Investig.

[32] Flórez CAR, García-Perdomo HA, Escudero MM (2021). MicroRNAs Associated with overweight and obesity in Childhood: a systematic review. MicroRNA.

[33] Kiran S, Kumar V, Kumar S, Price RL, Singh UP, Adipocyte (2021). Immune cells, and miRNA crosstalk: a Novel Regulator of metabolic dysfunction and obesity. Cells.

[34] Song M, Yu J, Li B, Dong J, Gao J, Shang L (2022). Identification of functionally important miRNA targeted genes associated with child obesity trait in genome-wide association studies. BMC Genomics.

[35] Gottmann P, Ouni M, Zellner L, Jähnert M, Rittig K, Walther D (2020). Polymorphisms in miRNA binding sites involved in metabolic diseases in mice and humans. Sci Rep.

[36] Mansego ML, Garcia-Lacarte M, Milagro FI, Marti A, Martinez JA (2017). DNA methylation of miRNA coding sequences putatively associated with childhood obesity. Pediatr Obes.

[37] Fontalba-Romero MI, Lopez-Enriquez S, Lago-Sampedro A, García-Escobar E, Pastori RL, Domínguez-Bendala J (2021). Association between the Mediterranean Diet and metabolic syndrome with serum levels of miRNA in morbid obesity. Nutrients.

[38] Youssef EM, Elfiky AM, BanglySoliman, Abu-Shahba N, Elhefnawi MM (2020). Expression profiling and analysis of some miRNAs in subcutaneous white adipose tissue during development of obesity. Genes Nutr.

[39] Altınkılıç EM, Bayrakdar S, Seymen Karabulut G, Haliloğlu B, Attar R (2022). The role of circulating miRNAs in leptin resistance in obese children. J Pediatr Endocrinol Metab.

[40] Feng X, Ding Y, Zhou M, Song N, Ding Y (2022). Integrative analysis of Exosomal miR-452 and miR-4713 Downregulating NPY1R for the Prevention of Childhood obesity. Dis Markers.

[41] Assmann TS, Cuevas-Sierra A, Riezu-Boj JI, Milagro FI, Martínez JA (2020). Comprehensive Analysis reveals novel interactions between circulating MicroRNAs and gut microbiota composition in human obesity. Int J Mol Sci.

[42] Guo Y, Zhu X, Zeng S, He M, Xing X, Wang C (2020). miRNA-10a-5p alleviates insulin resistance and maintains diurnal patterns of Triglycerides and Gut Microbiota in High-Fat Diet-Fed mice. Mediators Inflamm.

[43] Mennitti LV, Carpenter AAM, Loche E, Pantaleão LC, Fernandez-Twinn DS, Schoonejans JM (2022). Effects of maternal diet-induced obesity on metabolic disorders and age-associated miRNA expression in the liver of male mouse offspring. Int J Obes.

[44] Joshi A, Azuma R, Akumuo R, Goetzl L, Pinney SE (2020). Gestational diabetes and maternal obesity are associated with sex-specific changes in miRNA and target gene expression in the fetus. Int J Obes.

[45] Claycombe-Larson KG, Bundy AN, Roemmich JN (2020). Paternal high-fat diet and exercise regulate sperm miRNA and histone methylation to modify placental inflammation, nutrient transporter mRNA expression and fetal weight in a sex-dependent manner. J Nutr Biochem.

[46] Karere GM, Cox LA, Bishop AC, South AM, Shaltout HA, Mercado-Deane M-G (2021). Sex differences in MicroRNA expression and cardiometabolic risk factors in hispanic adolescents with obesity. J Pediatr.

[47] Lauria F, Iacomino G, Russo P, Venezia A, Marena P, Ahrens W (2022). Circulating miRNAs are Associated with inflammation biomarkers in children with overweight and obesity: results of the I.Family Study. Genes (Basel).

[48] Varghese M, Song J, Singer K (2021). Age and sex: impact on adipose tissue metabolism and inflammation. Mech Ageing Dev.

[49] Varghese M, Griffin C, Singer K. The role of sex and sex hormones in regulating Obesity-Induced inflammation. Adv Exp Med Biol. 2017;65–86. 10.1007/978-3-319-70178-3_5. 10.1007/978-3-319-70178-3_5 29224091

[50] ter Horst R, van den Munckhof ICL, Schraa K, Aguirre-Gamboa R, Jaeger M, Smeekens SP (2020). Sex-specific regulation of inflammation and metabolic syndrome in obesity. Arterioscler Thromb Vasc Biol.

[51] Rathod KS, Kapil V, Velmurugan S, Khambata RS, Siddique U, Khan S (2016). Accelerated resolution of inflammation underlies sex differences in inflammatory responses in humans. J Clin Invest.

[52] Simoes E, Correia-Lima J, Sardas L, Storti F, Otani TZ dos, Vasques S. Sex dimorphism in inflammatory response to obesity in childhood. Int J Obes. 2021;45:879–87. 10.1038/s41366-021-00753-1. 10.1038/s41366-021-00753-1 PMC8005372 33526854

[53] Jones A, Danielson KM, Benton MC, Ziegler O, Shah R, Stubbs RS (2017). miRNA signatures of insulin resistance in obesity. Obesity.

[54] Al-Rawaf HA (2019). Circulating microRNAs and adipokines as markers of metabolic syndrome in adolescents with obesity. Clin Nutr.

[55] Lin H, Tas E, Børsheim E, Mercer KE. Circulating miRNA signatures Associated with insulin resistance in adolescents with obesity. Diabetes Metab Syndr Obes 2020;Volume 13:4929–39. 10.2147/DMSO.S273908. 10.2147/DMSO.S273908 PMC7735788 33328751

[56] Cui X, You L, Zhu L, Wang X, Zhou Y, Li Y (2018). Change in circulating microRNA profile of obese children indicates future risk of adult diabetes. Metabolism.

[57] Kim H, Bae Y-U, Lee H, Kim H, Jeon JS, Noh H (2020). Effect of diabetes on exosomal miRNA profile in patients with obesity. BMJ Open Diabetes Res Care.

[58] Oses M, Margareto Sanchez J, Portillo MP, Aguilera CM, Labayen I (2019). Circulating miRNAs as biomarkers of obesity and Obesity-Associated Comorbidities in Children and Adolescents: a systematic review. Nutrients.

[59] Earle A, Bessonny M, Benito J, Huang K, Parker H, Tyler E (2022). Urinary exosomal MicroRNAs as biomarkers for Obesity-Associated chronic kidney disease. J Clin Med.

[60] Mafi A, Aghadavod E, Mirhosseini N, Mobini M, Asemi Z (2019). The effects of expression of different microRNAs on insulin secretion and diabetic nephropathy progression. J Cell Physiol.

[61] Ait-Aissa K, Nguyen QM, Gabani M, Kassan A, Kumar S, Choi S-K (2020). MicroRNAs and obesity-induced endothelial dysfunction: key paradigms in molecular therapy. Cardiovasc Diabetol.

[62] Wei M, Gao X, Liu L, Li Z, Wan Z, Dong Y (2020). Visceral adipose tissue derived Exosomes Exacerbate Colitis Severity *via* pro-inflammatory MiRNAs in high Fat Diet Fed mice. ACS Nano.

[63] Dogan H, Shu J, Hakguder Z, Xu Z, Cui J (2020). Elucidation of molecular links between obesity and cancer through microRNA regulation. BMC Med Genomics.

[64] Heyn GS, Corrêa LH, Magalhães KG. The impact of adipose tissue–derived miRNAs in metabolic syndrome, obesity, and Cancer. Front Endocrinol (Lausanne). 2020;11. 10.3389/fendo.2020.563816. 10.3389/fendo.2020.563816 PMC7573351 33123088

[65] Catanzaro G, Filardi T, Sabato C, Vacca A, Migliaccio S, Morano S (2021). Tissue and circulating microRNAs as biomarkers of response to obesity treatment strategies. J Endocrinol Invest.

[66] Ojeda-Rodríguez A, Assmann TS, Alonso‐Pedrero L, Azcona‐Sanjulian MC, Milagro FI, Marti A. Circulating < scp > miRNAs in girls with abdominal obesity: <scp > miR ‐221‐3p as a biomarker of response to weight loss interventions</scp >. Pediatr Obes. 2022;17. 10.1111/ijpo.12910. 10.1111/ijpo.12910 PMC9539627 35289984

[67] Assmann TS, Riezu-Boj JI, Milagro FI, Martínez JA (2020). Circulating adiposity‐related microRNAs as predictors of the response to a low‐fat diet in subjects with obesity. J Cell Mol Med.

[68] Liao J, Huang J, Wang S, Xiang M, Wang D, Deng H (2021). Effects of exercise and diet intervention on appetite-regulating hormones associated with miRNAs in obese children. Eating and Weight Disorders - Studies on Anorexia. Bulimia and Obesity.

[69] Wang X, Chen S, Lv D, Li Z, Ren L, Zhu H (2021). Liraglutide suppresses obesity and promotes browning of white fat via miR-27b *in vivo* and *in vitro*. J Int Med Res.

[70] Campolo F, Catanzaro G, Venneri MA, Ferretti E, Besharat ZM (2022). MicroRNA loaded edible nanoparticles: an emerging personalized therapeutic approach for the treatment of obesity and metabolic disorders. Theranostics.

[71] Thibonnier M, Esau C, Ghosh S, Wargent E, Stocker C (2020). Metabolic and energetic benefits of microRNA-22 inhibition. BMJ Open Diabetes Res Care.

[72] Ying W, Riopel M, Bandyopadhyay G, Dong Y, Birmingham A, Seo JB (2017). Adipose tissue macrophage-derived exosomal miRNAs can modulate in vivo and. Vitro Insulin Sensitivity Cell.

[73] Castaño C, Kalko S, Novials A, Párrizas M. Obesity-associated exosomal miRNAs modulate glucose and lipid metabolism in mice. Proceedings of the National Academy of Sciences 2018;115:12158–63. 10.1073/pnas.1808855115. 10.1073/pnas.1808855115 PMC6275521 30429322

[74] Heo Y, Kim H, Lim J, Choi SS (2022). Adipocyte differentiation between obese and lean conditions depends on changes in miRNA expression. Sci Rep.

[75] Roy D, Modi A, Ghosh R, Ghosh R, Benito-León J (2022). Visceral adipose tissue Molecular Networks and Regulatory microRNA in Pediatric obesity: an in Silico Approach. Int J Mol Sci.

[76] Akiyoshi K, Boersma GJ, Johnson MD, Velasquez FC, Dunkerly-Eyring B, O’Brien S (2021). Role of miR-181c in Diet-induced obesity through regulation of lipid synthesis in liver. PLoS ONE.

[77] Zhou X, Yuan Y, Teng F, Li K, Luo S, Zhang P (2021). Obesity-induced upregulation of microRNA-183-5p promotes hepatic triglyceride accumulation by targeting the B-cell translocation gene 1. Life Sci.

[78] Wei Z, Qin X, Kang X, Zhou H, Wang S, Wei D (2020). MiR-142-3p inhibits adipogenic differentiation and autophagy in obesity through targeting KLF9. Mol Cell Endocrinol.

[79] Gjorgjieva M, Sobolewski C, Ay A-S, Abegg D, Correia de Sousa M, Portius D (2020). Genetic ablation of MiR-22 fosters Diet-Induced obesity and NAFLD Development. J Pers Med.

[80] Huang X, Chen J, Ren Y, Fan L, Xiang W, He X (2022). Exosomal miR-122 promotes adipogenesis and aggravates obesity through the VDR/SREBF1 axis. Obesity.

[81] Shen L, He J, Zhao Y, Niu L, Chen L, Tang G (2021). MicroRNA-126b-5p exacerbates development of adipose tissue and Diet-Induced obesity. Int J Mol Sci.

[82] Yu Z, Luo R, Li Y, Li X, Yang Z, Peng J, et al. ADAR1 inhibits adipogenesis and obesity by interacting with dicer to promote the maturation of miR-155-5P. J Cell Sci. 2022;135. 10.1242/jcs.259333. 10.1242/jcs.259333 35067718

[83] Dong M, Ye Y, Chen Z, Xiao T, Liu W, Hu F (2020). MicroRNA 182 is a novel negative Regulator of adipogenesis by targeting CCAAT/Enhancer-Binding protein α. Obesity.

[84] Xu Y, Chen X, Zhao C, Wang X, Cheng Y, Xi F (2021). MiR-99b-5p attenuates adipogenesis by targeting SCD1 and Lpin1 in 3T3-L1 cells. J Agric Food Chem.

[85] Lima VM, Liu J, Brandão BB, Lino CA, Balbino Silva CS, Ribeiro MAC (2021). miRNA-22 deletion limits white adipose expansion and activates brown fat to attenuate high-fat diet-induced fat mass accumulation. Metabolism.

[86] Man X, Hu N, Tan S, Tang H, Guo Y, Tang C (2020). Insulin receptor substrate-1 inhibits high‐fat diet‐induced obesity by browning of white adipose tissue through miR‐503. FASEB J.

[87] Benito-Vicente A, Jebari-Benslaiman S, Galicia-Garcia U, Larrea-Sebal A, Uribe KB, Martin C. Molecular mechanisms of lipotoxicity-induced pancreatic β-cell dysfunction. Int Rev Cell Mol Biol. 2021;357–402. 10.1016/bs.ircmb.2021.02.013. 10.1016/bs.ircmb.2021.02.013 33832653

[88] Singh A, Kukreti R, Saso L, Kukreti S (2022). Mechanistic insight into oxidative stress-triggered signaling pathways and type 2 diabetes. Molecules.

[89] Yaribeygi H, Farrokhi FR, Butler AE, Sahebkar A (2019). Insulin resistance: review of the underlying molecular mechanisms. J Cell Physiol.

[90] Park SY, Gautier J-F, Chon S (2021). Assessment of insulin secretion and insulin resistance in human. Diabetes Metab J.

[91] Ma Y, Murgia N, Liu Y, Li Z, Sirakawin C, Konovalov R (2022). Neuronal miR-29a protects from obesity in adult mice. Mol Metab.

[92] Zhang F, Ma D, Zhao W, Wang D, Liu T, Liu Y (2020). Obesity-induced overexpression of miR-802 impairs insulin transcription and secretion. Nat Commun.

[93] Pan C, Li M, Wang J, Chu X, Xiong J, Yang X (2022). miR-4431 targets TRIP10/PRKD1 and impairs glucose metabolism. J Diabetes Investig.

[94] Agbu P, Carthew RW (2021). MicroRNA-mediated regulation of glucose and lipid metabolism. Nat Rev Mol Cell Biol.

[95] Zheng H, Wan J, Shan Y, Song X, Jin J, Su Q (2021). MicroRNA-185-5p inhibits hepatic gluconeogenesis and reduces fasting blood glucose levels by suppressing G6Pase. Theranostics.

[96] Li L, Zuo H, Huang X, Shen T, Tang W, Zhang X, et al. Bone marrow macrophage-derived exosomal miR‐143‐5p contributes to insulin resistance in hepatocytes by repressing MKP5. Cell Prolif. 2021;54. 10.1111/cpr.13140. 10.1111/cpr.13140 PMC8666281 34647385

[97] Cheng X, Huang Y, Yang P, Bu L (2020). miR-383 ameliorates high glucose-induced β-cells apoptosis and hyperglycemia in high-fat induced diabetic mice. Life Sci.

[98] Lischka J, Schanzer A, Hojreh A, Ba-Ssalamah A, de Gier C, Valent I (2021). Circulating microRNAs 34a, 122, and 192 are linked to obesity-associated inflammation and metabolic disease in pediatric patients. Int J Obes.

[99] Cabiati M, Fontanini M, Giacomarra M, Politano G, Randazzo E, Peroni D (2022). Screening and identification of putative long non-coding RNA in childhood obesity: evaluation of their transcriptional levels. Biomedicines.

[100] Xiao Q-Z, Zhu L-J, Fu Z-Y, Guo X-R, Chi X. Obesity related microRNA-424 is regulated by TNF&alpha; in adipocytes. Mol Med Rep 2020;23:1–1. 10.3892/mmr.2020.11659. 10.3892/mmr.2020.11659 33179089

[101] Deng L, Wang R, Li H, Zhang C, Zhao L, Zhang M. miRNA-Gene Regulatory Network in Gnotobiotic mice stimulated by dysbiotic gut microbiota transplanted from a genetically obese child. Front Microbiol. 2019;10. 10.3389/fmicb.2019.01517. 10.3389/fmicb.2019.01517 PMC6624655 31333621

[102] Matz AJ, Qu L, Karlinsey K, Zhou B (2022). MicroRNA-regulated B cells in obesity. Immunometabolism.

[103] Hulsmans M, Van Dooren E, Mathieu C, Holvoet P (2012). Decrease of miR-146b-5p in Monocytes during obesity is Associated with loss of the anti-inflammatory but not insulin signaling action of Adiponectin. PLoS ONE.

[104] Macartney-Coxson D, Danielson K, Clapham J, Benton MC, Johnstone A, Jones A (2020). MicroRNA profiling in adipose before and after weight loss highlights the role of miR‐223‐3p and the NLRP3 inflammasome. Obesity.

[105] Ávila-Escalante ML, Coop-Gamas F, Cervantes-Rodríguez M, Méndez-Iturbide D, Aranda-González II. The effect of diet on oxidative stress and metabolic diseases—clinically controlled trials. J Food Biochem. 2020;1–16. 10.1111/jfbc.13191. 10.1111/jfbc.13191 32160647

[106] Ramazi S, Zahiri J. Post-translational modifications in proteins: resources, tools and prediction methods. Database 2021;2021. 10.1093/database/baab012. 10.1093/database/baab012 PMC8040245 33826699

[107] Magalhães A, Duarte HO, Reis CA (2021). The role of O-glycosylation in human disease. Mol Aspects Med.

[108] Gao Q, Exp Med B. ; 2019, p. 179–98. 10.1007/978-981-15-0602-4_9.

[109] Sharifi-Rad M, Anil Kumar NV, Zucca P, Varoni EM, Dini L, Panzarini E, et al. Lifestyle, oxidative stress, and Antioxidants: back and forth in the pathophysiology of Chronic Diseases. Front Physiol. 2020;11. 10.3389/fphys.2020.00694. 10.3389/fphys.2020.00694 PMC7347016 32714204

[110] Ge T, Yang J, Zhou S, Wang Y, Li Y, Tong X. The role of the Pentose phosphate pathway in diabetes and Cancer. Front Endocrinol (Lausanne). 2020;11. 10.3389/fendo.2020.00365. 10.3389/fendo.2020.00365 PMC7296058 32582032

[111] Tatone C, di Emidio G, Vitti M, di Carlo M, Santini S, D’Alessandro AM (2015). Sirtuin Functions in female fertility: possible role in oxidative stress and aging. Oxid Med Cell Longev.

[112] Görlach A, Dimova EY, Petry A, Martínez-Ruiz A, Hernansanz-Agustín P, Rolo AP (2015). Reactive oxygen species, nutrition, hypoxia and diseases: problems solved?. Redox Biol.

[113] Tobon-Velasco J, Cuevas E, Torres-Ramos M (2014). Receptor for AGEs (RAGE) as mediator of NF-kB pathway activation in neuroinflammation and oxidative stress. CNS Neurol Disord Drug Targets.

[114] Lingappan K (2018). NF-κB in oxidative stress. Curr Opin Toxicol.

[115] Peng Y, Kim J-M, Park H-S, Yang A, Islam C, Lakatta EG (2016). AGE-RAGE signal generates a specific NF-κB RelA barcode that directs collagen I expression. Sci Rep.

[116] Muzio G, Barrera G, Pizzimenti S (2021). Peroxisome proliferator-activated receptors (PPARs) and oxidative stress in physiological conditions and in Cancer. Antioxidants.

[117] Schrader M, Fahimi HD (2006). Peroxisomes and oxidative stress. Biochimica et Biophysica Acta (BBA). Mol Cell Res.

[118] Gonzalez-Dominguez A, Lechuga-Sancho AM, Gonzalez-Dominguez R (2018). Intervention and observational trials are complementary in Metabolomics: diabetes and the oral glucose tolerance test. Curr Top Med Chem.

[119] Yan LJ. Pathogenesis of chronic hyperglycemia: From reductive stress to oxidative stress. J Diabetes Res 2014;2014. 10.1155/2014/137919. 10.1155/2014/137919 PMC4082845 25019091

[120] Yamagishi SI (2013). Advanced Glycation End-Products. Brenner’s Encyclopedia of Genetics. Second Ed.

[121] Yaribeygi H, Atkin SL, Sahebkar A (2019). A review of the molecular mechanisms of hyperglycemia-induced free radical generation leading to oxidative stress. J Cell Physiol.

[122] Gao L, Mann GE (2009). Vascular NAD(P)H oxidase activation in diabetes: a double-edged sword in redox signalling. Cardiovasc Res.

[123] Fakhruddin S, Alanazi W, Jackson KE. Diabetes-Induced Reactive Oxygen Species: Mechanism of Their Generation and Role in Renal Injury. J Diabetes Res 2017;2017. 10.1155/2017/8379327. 10.1155/2017/8379327 PMC5253173 28164134

[124] Ma C-H, Wu C-H, Jou I-M, Tu Y-K, Hung C-H, Hsieh P-L (2018). PKR activation causes inflammation and MMP-13 secretion in human degenerated articular chondrocytes. Redox Biol.

[125] Santillan LD, Olivera Vargas JA, Gomez Mejiba SE, Gimenez MS, Siewert SE, Ramirez DC (2017). Rationale for Targeting Nrf2 to reduce the metabolic risk: a study in overweight boys and rats Fed a Hypercaloric Diet. Free Radic Biol Med.

[126] Akyürek N, Aycan Z, Çetinkaya S, Akyürek Ö, Yilmaz Ağladioğlu S, Ertan Ü (2013). Peroxisome proliferator activated receptor (PPAR)-gamma concentrations in childhood obesity. Scand J Clin Lab Invest.

[127] Arab Sadeghabadi Z, Nourbakhsh M, Pasalar P, Emamgholipour S, Golestani A, Larijani B (2018). Reduced gene expression of sirtuins and active AMPK levels in children and adolescents with obesity and insulin resistance. Obes Res Clin Pract.

[128] Gastaldi G, Russell A, Golay A, Giacobino J-P, Habicht F, Barthassat V (2007). Upregulation of peroxisome proliferator-activated receptor gamma coactivator gene (PGC1A) during weight loss is related to insulin sensitivity but not to energy expenditure. Diabetologia.

[129] Darroudi S, Fereydouni N, Tayefi M, Ahmadnezhad M, Zamani P, Tayefi B (2019). Oxidative stress and inflammation, two features associated with a high percentage body fat, and that may lead to diabetes mellitus and metabolic syndrome. BioFactors.

[130] Codoñer-Franch P, Valls-Bellés V, Arilla-Codoñer A, Alonso-Iglesias E (2011). Oxidant mechanisms in childhood obesity: the link between inflammation and oxidative stress. Translational Res.

[131] Lee J-H, Go Y, Kim D-Y, Lee SH, Kim O-H, Jeon YH (2020). Isocitrate dehydrogenase 2 protects mice from high-fat diet-induced metabolic stress by limiting oxidative damage to the mitochondria from brown adipose tissue. Exp Mol Med.

[132] Włodarski A, Strycharz J, Wróblewski A, Kasznicki J, Drzewoski J, Śliwińska A (2020). The role of microRNAs in metabolic syndrome-related oxidative stress. Int J Mol Sci.

[133] Klisic A, Radoman Vujacic I, Munjas J, Ninic A, Kotur-Stevuljevic J (2022). Micro-ribonucleic acid modulation with oxidative stress and inflammation in patients with type 2 diabetes mellitus – a review article. Archives of Medical Science.

[134] Ruiz GP, Camara H, Fazolini NPB, Mori MA (2021). Extracellular miRNAs in redox signaling: Health, disease and potential therapies. Free Radic Biol Med.

[135] Robson A (2020). Oxidation of miRNAs by ROS leads to cardiac hypertrophy. Nat Rev Cardiol.

[136] Wang J-X, Gao J, Ding S-L, Wang K, Jiao J-Q, Wang Y (2015). Oxidative modification of miR-184 enables it to target Bcl-xL and Bcl-w. Mol Cell.

[137] Seok H, Lee H, Lee S, Ahn SH, Lee H-S, Kim G-WD (2020). Position-specific oxidation of miR-1 encodes cardiac hypertrophy. Nature.

[138] Murri M, el Azzouzi H (2018). MicroRNAs as regulators of mitochondrial dysfunction and obesity. Am J Physiol Heart Circ Physiol.

[139] Ji J, Qin Y, Ren J, Lu C, Wang R, Dai X (2015). Mitochondria-related mir-141-3p contributes to mitochondrial dysfunction in HFD-induced obesity by inhibiting PTEN. Sci Rep.

[140] Christian P, Su Q (2014). MicroRNA regulation of mitochondrial and ER stress signaling pathways: implications for lipoprotein metabolism in metabolic syndrome. Am J Physiology-Endocrinology Metabolism.

[141] Liu H, Mao Z, Zhu J, Shen M, Chen F (2020). MiR-140-5p inhibits oxidized low-density lipoprotein-induced oxidative stress and cell apoptosis via targeting toll-like receptor 4. Gene Ther.

[142] Qin S-B, Peng D-Y, Lu J-M, Ke Z-P (2018). MiR-182-5p inhibited oxidative stress and apoptosis triggered by oxidized low-density lipoprotein via targeting toll-like receptor 4. J Cell Physiol.

[143] Zhuang X, Li R, Maimaitijiang A, Liu R, Yan F, Hu H (2019). Mir-221‐3p inhibits oxidized low‐density lipoprotein induced oxidative stress and apoptosis via targeting a disintegrin and metalloprotease‐22. J Cell Biochem.

[144] Lo W-Y, Yang W-K, Peng C-T, Pai W-Y, Wang H-J. MicroRNA-200a/200b modulate high Glucose-Induced endothelial inflammation by targeting O-linked N-Acetylglucosamine transferase expression. Front Physiol 2018;9. 10.3389/fphys.2018.00355. 10.3389/fphys.2018.00355 PMC5915961 29720943

[145] Rovira-Llopis S, Díaz-Rúa R, Grau-del Valle C, Iannantuoni F, Abad-Jimenez Z, Bosch-Sierra N (2021). Characterization of differentially expressed circulating miRNAs in metabolically healthy versus unhealthy obesity. Biomedicines.

[146] Infante-Menéndez J, López-Pastor AR, González-López P, Gómez-Hernández A, Escribano O (2020). The interplay between oxidative stress and miRNAs in Obesity-Associated hepatic and vascular complications. Antioxidants.

[147] Varghese JF, Patel R, Yadav UCS (2018). Novel insights in the metabolic syndrome-induced oxidative stress and inflammation-mediated atherosclerosis. Curr Cardiol Rev.

[148] Li Y, Zhou Q, Pei C, Liu B, Li M, Fang L (2016). Hyperglycemia and Advanced Glycation End Products regulate miR-126 expression in endothelial progenitor cells. J Vasc Res.

[149] Chen Q, Shen Z, Mao Y, Li Q, Liu Y, Mei M (2019). Inhibition of microRNA-34a mediates protection of thymosin beta 4 in endothelial progenitor cells against advanced glycation endproducts by targeting B-cell lymphoma 2. Can J Physiol Pharmacol.

[150] Varga ZV, Kupai K, Szűcs G, Gáspár R, Pálóczi J, Faragó N (2013). MicroRNA-25-dependent up-regulation of NADPH oxidase 4 (NOX4) mediates hypercholesterolemia-induced oxidative/nitrative stress and subsequent dysfunction in the heart. J Mol Cell Cardiol.

[151] Wang H-J, Huang Y-L, Shih Y-Y, Wu H-Y, Peng C-T, Lo W-Y (2014). MicroRNA-146a decreases high Glucose/Thrombin-Induced endothelial inflammation by inhibiting NAPDH oxidase 4 expression. Mediators Inflamm.

[152] La Sala L, Mrakic-Sposta S, Micheloni S, Prattichizzo F, Ceriello A (2018). Glucose-sensing microRNA-21 disrupts ROS homeostasis and impairs antioxidant responses in cellular glucose variability. Cardiovasc Diabetol.

[153] Hu B, Gong Z, Bi Z. Inhibition of miR-383 suppresses oxidative stress and improves endothelial function by increasing sirtuin 1. Braz J Med Biol Res 2020;53. 10.1590/1414-431x20198616. 10.1590/1414-431X20198616 PMC6984384 31994599

[154] Kong X, Liu C, Wang G, Yang H, Yao X, Hua Q (2019). LncRNA LEGLTBC Functions as a ceRNA to antagonize the Effects of miR-34a on the downregulation of SIRT1 in Glucolipotoxicity-Induced INS-1 Beta cell oxidative stress and apoptosis. Oxid Med Cell Longev.

[155] Briones-Espinoza MJ, Cortés-García JD, Vega-Cárdenas M, Uresti-Rivera EU, Gómez-Otero A, López-López N (2020). Decreased levels and activity of Sirt1 are modulated by increased miR-34a expression in adipose tissue mononuclear cells from subjects with overweight and obesity: a pilot study. Diabetes & Metabolic Syndrome: Clinical Research & Reviews.

[156] Portius D, Sobolewski C, Foti M (2017). MicroRNAs-Dependent regulation of PPARs in metabolic Diseases and Cancers. PPAR Res.

[157] Jennewein C, von Knethen A, Schmid T, Brüne B (2010). MicroRNA-27b contributes to Lipopolysaccharide-mediated peroxisome proliferator-activated receptor γ (PPARγ) mRNA destabilization. J Biol Chem.

[158] Azzimato V, Chen P, Barreby E, Morgantini C, Levi L, Vankova A (2021). Hepatic miR-144 drives fumarase activity preventing NRF2 activation during obesity. Gastroenterology.

[159] Engedal N, Žerovnik E, Rudov A, Galli F, Olivieri F, Procopio AD (2018). From oxidative stress damage to Pathways, Networks, and Autophagy via MicroRNAs. Oxid Med Cell Longev.

[160] Hanousková B, Vávrová G, Ambrož M, Boušová I, Karlsen TA, Skálová L (2021). MicroRNAs mediated regulation of glutathione peroxidase 7 expression and its changes during adipogenesis. Biochimica et Biophysica Acta (BBA). Gene Regul Mech.

[161] la Sala L, Cattaneo M, de Nigris V, Pujadas G, Testa R, Bonfigli AR (2016). Oscillating glucose induces microRNA-185 and impairs an efficient antioxidant response in human endothelial cells. Cardiovasc Diabetol.

[162] la Sala L, Crestani M, Garavelli S, de Candia P, Pontiroli AE (2020). Does microRNA Perturbation Control the Mechanisms linking obesity and diabetes? Implications for Cardiovascular Risk. Int J Mol Sci.

[163] Xu G, Chen J, Jing G, Shalev A (2013). Thioredoxin-interacting protein regulates insulin transcription through microRNA-204. Nat Med.

[164] He C, Yang J, Ding J, Li S, Wu H, Xiong Y (2018). Downregulation of glucose-6phosphate dehydrogenase by microRNA-1 inhibits the growth of pituitary tumor cells. Oncol Rep.

[165] Zheng W, Feng Q, Liu J, Guo Y, Gao L, Li R, et al. Inhibition of 6-phosphogluconate dehydrogenase reverses Cisplatin Resistance in Ovarian and Lung Cancer. Front Pharmacol. 2017;8. 10.3389/fphar.2017.00421. 10.3389/fphar.2017.00421 PMC5491617 28713273

[166] Qiu Z, Guo W, Wang Q, Chen Z, Huang S, Zhao F (2015). MicroRNA-124 reduces the pentose phosphate pathway and proliferation by targeting PRPS1 and RPIA mRNAs in human colorectal Cancer cells. Gastroenterology.

[167] Fu X, Huang X, Li P, Chen W, Xia M (2014). 7-Ketocholesterol inhibits isocitrate dehydrogenase 2 expression and impairs endothelial function via microRNA-144. Free Radic Biol Med.

[168] Forman HJ, Zhang H (2021). Targeting oxidative stress in disease: promise and limitations of antioxidant therapy. Nat Rev Drug Discov.

[169] Wang J, Liao B, Wang C, Zhong O, Lei X, Yang Y (2022). Effects of antioxidant supplementation on metabolic Disorders in obese patients from Randomized Clinical Controls: a Meta-analysis and systematic review. Oxid Med Cell Longev.

[170] Cannataro R, Caroleo MC, Fazio A, La Torre C, Plastina P, Gallelli L (2019). Ketogenic Diet and microRNAs linked to antioxidant biochemical homeostasis. Antioxidants.

[171] Corrêa TA, Rogero MM (2019). Polyphenols regulating microRNAs and inflammation biomarkers in obesity. Nutrition.

[172] Bladé C, Baselga-Escudero L, Salvadó MJ, Arola-Arnal A (2013). miRNAs, polyphenols, and chronic disease. Mol Nutr Food Res.

[173] Ahn J, Lee H, Jung CH, Ha T (2012). Lycopene inhibits hepatic steatosis via microRNA-21-induced downregulation of fatty acid-binding protein 7 in mice fed a high-fat diet. Mol Nutr Food Res.

[174] Miura A, Ikeda A, Abe M, Seo K, Watanabe T, Ozaki-Masuzawa Y (2021). Diallyl Trisulfide prevents obesity and decreases miRNA‐335 expression in adipose tissue in a Diet‐Induced obesity rat model. Mol Nutr Food Res.

[175] Milenkovic D, Jude B, Morand C (2013). miRNA as molecular target of polyphenols underlying their biological effects. Free Radic Biol Med.

[176] Valko M, Jomova K, Rhodes CJ, Kuča K, Musílek K (2016). Redox- and non-redox-metal-induced formation of free radicals and their role in human disease. Arch Toxicol.

[177] González-Domínguez Á, Millán-Martínez M, Domínguez-Riscart J, Mateos RM, Lechuga-Sancho AM, González-Domínguez R (2022). Altered metal Homeostasis Associates with inflammation, oxidative stress, impaired glucose metabolism, and Dyslipidemia in the crosstalk between Childhood obesity and insulin resistance. Antioxidants.

[178] Fan Y, Zhang C, Bu J (2017). Relationship between selected serum metallic elements and obesity in children and adolescent in the U.S. Nutrients.

[179] Błażewicz A, Klatka M, Astel A, Partyka M, Kocjan R (2013). Differences in Trace metal concentrations (Co, Cu, Fe, Mn, Zn, Cd, and Ni) in whole blood, plasma, and urine of obese and nonobese children. Biol Trace Elem Res.

[180] González-Domínguez Á, Millán-Martínez M, Sánchez-Rodas D, Lechuga-Sancho AM, González-Domínguez R. Characterization of the plasmatic and erythroid Multielemental Biodistribution in Childhood obesity using a high-throughput method for size fractionation of metal species, 2023, p. 123–32. 10.1007/978-1-0716-2699-3_12. 10.1007/978-1-0716-2699-3_12 36152156

[181] González-Domínguez Á, Domínguez-Riscart J, Millán-Martínez M, Lechuga-Sancho AM, González-Domínguez R. Exploring the association between circulating trace elements, metabolic risk factors, and the adherence to a Mediterranean diet among children and adolescents with obesity. Front Public Health. 2023;10. 10.3389/fpubh.2022.1016819. 10.3389/fpubh.2022.1016819 PMC9880061 36711380

[182] González-Domínguez Á, Domínguez‐Riscart J, Millán‐Martínez M, Mateos‐Bernal RM, Lechuga‐Sancho AM, González‐Domínguez R. Trace elements as potential modulators of puberty‐induced amelioration of oxidative stress and inflammation in childhood obesity. BioFactors 2023. 10.1002/biof.1946. 10.1002/biof.1946 36929162

[183] González-Domínguez Á, Domínguez‐Riscart J, Millán‐Martínez M, Lechuga‐Sancho AM, González‐Domínguez R (2023). Sexually dimorphic metal alterations in childhood obesity are modulated by a complex interplay between inflammation, insulin, and sex hormones. BioFactors.

[184] González-Domínguez Á, Visiedo-García FM, Domínguez-Riscart J, González-Domínguez R, Mateos RM, Lechuga-Sancho AM (2020). Iron metabolism in obesity and metabolic syndrome. Int J Mol Sci.

[185] Barr I, Smith AT, Chen Y, Senturia R, Burstyn JN, Guo F (2012). Ferric, not ferrous, heme activates RNA-binding protein DGCR8 for primary microRNA processing. Proc Natl Acad Sci.

[186] Faller M, Matsunaga M, Yin S, Loo JA, Guo F (2007). Heme is involved in microRNA processing. Nat Struct Mol Biol.

[187] Weitz SH, Gong M, Barr I, Weiss S, Guo F. Processing of microRNA primary transcripts requires heme in mammalian cells. Proceedings of the National Academy of Sciences 2014;111:1861–6. 10.1073/pnas.1309915111. 10.1073/pnas.1309915111 PMC3918773 24449907

[188] Nguyen TA, Park J, Dang TL, Choi Y-G, Kim VN (2018). Microprocessor depends on hemin to recognize the apical loop of primary microRNA. Nucleic Acids Res.

[189] Yuan Q, Zhang Z, Hu X, Liao J, Kuang J (2019). miR-374a/Myc axis modulates iron overload-induced production of ROS and the activation of hepatic stellate cells via TGF-β1 and IL-6. Biochem Biophys Res Commun.

[190] Zhao X, Si L, Bian J, Pan C, Guo W, Qin P (2022). Adipose tissue macrophage-derived exosomes induce ferroptosis via glutathione synthesis inhibition by targeting SLC7A11 in obesity-induced cardiac injury. Free Radic Biol Med.

[191] Rivkin M, Simerzin A, Zorde-Khvalevsky E, Chai C, Yuval JB, Rosenberg N (2016). Inflammation-Induced expression and secretion of MicroRNA 122 leads to reduced blood levels of kidney-derived erythropoietin and Anemia. Gastroenterology.

[192] Pulkkinen KH, Ylä-Herttuala S, Levonen A-L (2011). Heme oxygenase 1 is induced by miR-155 via reduced BACH1 translation in endothelial cells. Free Radic Biol Med.

[193] Hou W, Tian Q, Steuerwald NM, Schrum LW, Bonkovsky HL (2012). The let-7 microRNA enhances heme oxygenase-1 by suppressing Bach1 and attenuates oxidant injury in human hepatocytes. Biochimica et Biophysica Acta (BBA). Gene Regul Mech.

[194] Kabaria S, Choi DC, Chaudhuri AD, Jain MR, Li H, Junn E (2015). MicroRNA-7 activates Nrf2 pathway by targeting Keap1 expression. Free Radic Biol Med.

[195] Gou L, Zhao L, Song W, Wang L, Liu J, Zhang H (2018). Inhibition of miR-92a suppresses oxidative stress and improves endothelial function by upregulating Heme Oxygenase-1 in db/db mice. Antioxid Redox Signal.

[196] Chang C-L, Au L-C, Huang S-W, Fai Kwok C, Ho L-T, Juan C-C (2011). Insulin Up-Regulates Heme Oxygenase-1 expression in 3T3-L1 adipocytes via PI3-Kinase- and PKC-Dependent pathways and Heme Oxygenase-1–Associated MicroRNA downregulation. Endocrinology.

[197] Meerson A, Yehuda H (2016). Leptin and insulin up-regulate miR-4443 to suppress NCOA1 and TRAF4, and decrease the invasiveness of human colon cancer cells. BMC Cancer.

[198] Blahna MT, Hata A (2012). Smad-mediated regulation of microRNA biosynthesis. FEBS Lett.

[199] Lu S, Mott JL, Harrison-Findik DD (2015). Saturated fatty acids induce post-transcriptional regulation of HAMP mRNA via AU-rich element-binding protein, Human Antigen R (HuR). J Biol Chem.

